# Remotely Sensed Image Classification by Complex Network Eigenvalue and Connected Degree

**DOI:** 10.1155/2012/632703

**Published:** 2012-01-02

**Authors:** Mengxi Xu, Chenglin Wei

**Affiliations:** ^1^School of Computer Engineering, Nanjing Institute of Technology, Nanjing 211167, China; ^2^Nanjing Rail Traffic Technology Company, Department of Electrical and Mechanical Control, NARI Technology Development Co., Ltd., Nanjing 210061, China

## Abstract

It is a well-known problem of remotely sensed images classification due to its complexity. This paper proposes a remotely sensed image classification method based on weighted complex network clustering using the traditional *K*-means clustering algorithm. First, the degree of complex network and clustering coefficient of weighted feature are used to extract the features of the remote sensing image. Then, the integrated features of remote sensing image are combined to be used as the basis of classification. Finally, *K*-means algorithm is used to classify the remotely sensed images. The advantage of the proposed classification method lies in obtaining better clustering centers. The experimental results show that the proposed method gives an increase of 8% in accuracy compared with the traditional *K*-means algorithm and the Iterative Self-Organizing Data Analysis Technique (ISODATA) algorithm.

## 1. Introduction

Remote sensing is an all-round detective technology rose and rapidly developed from 1960s, which shows its superiority in urban planning, resources exploration, environment protection, land monitoring, agriculture, forestry, military and so on, and still develops its applications in breadth and depth.

Remotely sensed image classification is an important issue in remote sensing technique applications, whose goal is to classify the pixels in the remotely sensed images by ground-object categories. For example, the images are divided into many districts which represent forest, grass, lake, town, and other ground-object categories. Remotely sensed image classification can be carried on according to the following steps. First, feature parameters are analyzed and chosen according to the spectral characteristics of each ground-object. Second, feature space is divided into nonoverlapping subspaces. Then, each pixel vector in the images is assigned to each sub-space.

The classification of remotely sensed image is divided into supervised classification and unsupervised classification. The basic principle of supervised classification is to determine discriminant functions and corresponding criterion according to prior knowledge of classification, and the progress is to determine undetermined parameters in discriminant functions by taking advantage of a certain amount of samples' observed values in known classifications, which is called learning, then, the samples' observed values of unknown classification are put into discriminant functions, and the sample's classification is determined according to the criterion. There are several kinds of commonly used supervised classification approaches. Minimum range classification is to use the distance in feature space to express element data and the similar degree of classified category characteristic. After each category characteristic parameter by the training data is obtained, the distance between the unknown element and each eigenvector or eigenvector represented is first calculated, and then the unknown element is assigned to the category with the least distance. Maximum likelihood classification is to calculate the likelihood of each pixel point by point and this pixel is assigned to the category corresponding to the maximum likelihood. This classification precision is high, but the assorting process is complex and the computing time is long. Parallelepiped classification carries on the classification with a simple decision rule to the remotely sensed data. Decision boundary in the image data space forms an *N*-dimensional parallelepiped. Parallelepiped's dimension is determined by the threshold value of standard deviation of classification's mean value for each category. If the element is between the low threshold value and the high threshold value for *N* bands, then it belongs to this kind. If the element value falls in many kinds, then this element will turn over to the last match type. Decision tree classification follows the guiding principle of the hierarchical classification idea. The hierarchical classification idea aims at each kind of ground-object's different information characteristic and decomposes it according to certain principle layer by layer. In each decomposition process, according to the different subdomain characteristic and the prior knowledge, the researcher may choose the different bands or the band combination for classification. Based on images' different characteristics, the decision tree classification produces the rule and discovers the law by the tree structure expression classification or the decision-making set. First, discriminant function is produced using the training space entity set. Second, lower level points and branches in each branch subset are repeatedly established according to different values. Finally, the decision tree is built. This method is flexible, intuitive, clear, vigorous, and healthy and the operation efficiency is high. The decision tree classification approach has highlighted vegetation's spatial distribution, particularly the sparse vegetation's spatial distribution, and the vegetation and the nonvegetation region might use the threshold value to separate, which increases the classified precision.

The nonsupervised classification [1–3] refers to the assorting process without exerting any prior knowledge and carries on the classification naturally according to the remote sensing spectrum's characteristic. The commonly used nonsupervised classification approaches mainly include the *K*-means algorithm and the ISODATA algorithm.

The *K*-means algorithm's basic philosophy is through the iteration which moves each kind of classifications' centers until obtaining the best cluster result. The ISODATA sorting algorithm clusters the element data with the smallest spectrum formula. It starts from the random cluster mean value, computes the distance of the element and the mean value, and assigns the individual to the recent category. Every time the cluster repeats, the cluster mean value changes one time, and the new mean value is taken as the next-time cluster circulation. The ISODATA cluster is finished until either the greatest number is repeated or the largest percentage of invariable element between two repeated times is achieved.

The *K*-means algorithm and the ISODATA algorithm mainly have two different aspects: (1) each time the *K*-means algorithm adjusts a category of sample, each kind of sample's average value is recalculated, which is called one-by-one sample correction method. Instead, the ISODATA algorithm recalculates each kind of sample's average value after adjusting all samples, which is called batches of sample classifications; (2) the *K*-means algorithm may complete the sample cluster analysis by adjusting sample attribute category, but the ISODATA algorithm can not only complete the sample cluster analysis through the adjustment of sample attribute category, but also automatically carry on the merge and the fission of the category, which has a quite reasonable cluster result of the class number.

The above classification approaches have been used for the mid- and low-resolution satellite remotely sensed image, which processes high spectrum in the universal applications, while the high-resolution remotely sensed image actually has many insufficiencies now.

This paper proposes one kind of new remotely sensed image classification method by the combination of the complex network architecture characteristic and the *K*-means cluster algorithm idea. The proposed algorithm constructs the weighted network synthesis characteristic value according to the similarity formula and then classifies by the *K*-means cluster algorithm's idea to find the most superior category division.

In this paper, the first section is the introduction. The main principle of the complex network is briefly outlined in the second section, the remotely sensed image classification method based on complex network is proposed in the third section, and the fourth section shows simulated experiments and results analysis. Finally, the fifth section gives the conclusion and prospect.

## 2. Complex Networks

Since the end of 20th century, complex network [4, 5] has emerged gradually and overlapped rapidly with other disciplines in the depth and the breadth. Specially, two groundbreaking international works have started not a small upsurge of researching complex network. First, Watts and Strogatz [6] published the article in the Nature magazine in 1998, which introduced the microcosm network model, and described the transformation from the completely regular network to the completely random network. The microcosm network both has cluster characteristic similar to the regular network and small average path length similar to the random network. Second, Barabasi and Albert [7] published the article in *Science* to point out that many actual complex network's connection distributions have the power law form in 1999. As the power law distribution has no obvious characteristic length, this kind of network is called the scale-free network [8]. Then the scientists have studied each characteristic of each kind of complex network. The domestic researchers also already noted this tendency and also started to launch the research. The scholars joining the complex network research mainly come from domains such as graph theory, statistical physics, computer network research, ecology, sociology as well as economics. The networks involved mainly include networks in life sciences domain (e.g., cellular network, protein-protein function network, protein fold network, neural network, ecology networks), the Internet/WWW networks, the social networks which include the dissemination network of the epidemic disease, the scientist cooperative network, human sexual relationship network, and linguistics network. The main methods used in the networks are graph theory in mathematics, the statistical physics method in physics, and the society network analysis method. However, except the example of medical images applications [9], the complex network analysis is not applied in the remotely sensed image classification in domestic [10].

### 2.1. Complex Network and Key Characteristics

The statistical properties of the complex network architecture mainly have the average path length, the clustering coefficient, degree distribution, and betweenness.

#### 2.1.1. Average Path Length

In the network, the distance *d*_*ij*_ between the two nodes *i* and *j* is defined as

(1) $$d_{ij}=\sqrt{{\left(i-j\right)}^{T}\left(i-j\right).}$$

The average path length *L* in the network is defined as the mean value of the distance between the random two nodes, namely,

(2) $$L=\frac{1}{\left(1/2\right)N\left(N-1\right)}\underset{i\geq j}{\sum }d_{ij},$$

where *N* is the number of the network nodes. The average path length of the network is also called the network characteristic path length [11].

#### 2.1.2. Clustering Coefficients

Generally, assume the node *i* in the network has *n* edges to be connected with other *n* nodes, then these *n* nodes are called the neighbors of the node *i*. Obviously, there are *n*(*n* − 1)/2 edges at most. But between these *n* nodes, the ratio of the actually existing *m* edges and the possibly existing edges is defined as the clustering coefficient of node *i*, namely,

(3) $$C=\frac{2m}{n\left(n-1\right)}.$$

#### 2.1.3. Distribution among Degrees

Degree is a simple and important concept in the independent node attribute. Node *i* is defined as another node number connecting this node. In network the mean value of all nodes' degrees is called the network average degree and is recorded as *k*. In network distributed situation of nodes' degrees can be described by available distribution function *P*(*k*), which expresses the probability that the randomly designated node's degree is *k*.

#### 2.1.4. Betweenness

In the complex network, there are some nodes which are not very large, but they are actually significant like a bridge in the entire structure [12]. The betweenness of the nodes has reflected the bridge ability size of the nodes.

Let *n*_*st*_ expresses the number of the most short path from apex *s* to *t*, the *n*_*st*_(*i*) expresses apex *i*'s number of the most short path from *s* to *t*, then the betweenness of the node *i* is

(4) $$C_{B}\left(v\right)=\underset{s\neq t\neq i\in V}{\sum }\frac{n_{st}\left(i\right)}{n_{st}}.$$

Node accumulation coefficient [13] has reflected the network module nature. Namely, the interconnection of the interior node in the identical module is high, while the accumulation is strong, but the node accumulation coefficient between modules is weak. The node accumulation coefficient manifests the local interconnection density of this node, but for the complex network, the node's connection represents some similarity in the node attribute, therefore, the accumulation degree and the accumulation coefficient can be used as the characteristic to cluster the network node.

### 2.2. Weighted Complex Network and Its Key Characteristics

At present the research for complex network mainly aims at the unweighted complex network. But in the realistic network, the weights of the edges are often dissimilar and will affect the performance of the entire network. The weighted complex network can better express the structure of the complex network than unweighted complex network. Comparing to the definition of the degree and the accumulation coefficients above, the definition of the nodes' weighted degree, the weighted accumulation, and the weighted accumulation, coefficient below in the weighted complex network are given [4].

#### 2.2.1. Weighted Degree

A node's degree may be defined as the sum of the weights of this node and its all neighboring nodes, which is also called weighted degree, namely, the weighted degree WD_*i*_ of the node *i* is defined as follows:

(5) $$\text{W}{\text{D}}_{i}=\sum w_{ij},$$

where *w*_*ij*_  (*w*_*ij*_ > 0) expresses the weighted coefficients between node *i* and *j*.

The node's weighted degree reflects the joint strength between this node and other nodes. The larger the node's weighted degree, the more this node is suitable to be the cluster center.

#### 2.2.2. Weighted Clustering Coefficient

The clustering coefficient WC_*i*_ of the node *i* is

(6) $$\text{W}{\text{C}}_{i}=\frac{2\sum w_{jk}}{D_{i}\left(D_{i}-1\right)},$$

where *D*_*i*_  express the degree of node  *i*, which is defined as number of edges that is connected with node  *i*, and *j* and *k* are neighboring nodes of node *i*.

The node's weighted clustering coefficient manifests local interconnection density and the intensity of this node. The larger the node's weighted clustering coefficient is, the more the node is suitable to be the cluster center.

#### 2.2.3. Weighted Network Synthesis Characteristic Value

The weighted network synthesis characteristic value of the node *i* is

(7) $$\text{WC}F_{i}=a\text{W}{\text{C}}_{i}+\frac{\left(1-a\right)\text{W}{\text{D}}_{i}}{N},$$

where *N* is the node number in the network, *a* is the adjustable parameter, 0 < *a* < 1. Regarding the different application background, *a* takes the different value, which indicates that weighted degree and the weighted clustering coefficient occupy different proportion in the weighted network synthesis characteristic value. The larger the *a* is, the bigger proportion the weighted clustering coefficient in the weighted network synthesis characteristic value occupies, and the smaller the *a* is, the bigger proportion the weighted degree in the weighted network synthesis characteristic value occupies.

#### 2.2.4. Connected Degree

The connection *r*_*ij*_ between the node *i* and *j* in complex network is

(8) $$r_{ij}=\frac{\langle ij\rangle -\langle i\rangle \langle j\rangle }{{\left[\left(\langle i^{2}\rangle -{\langle i\rangle }^{2}\right)\left(\langle j^{2}\rangle {\langle j\rangle }^{2}\right)\right]}^{1/2}},$$

where 〈*i*〉 expresses the mean value to corresponding element in node *i*. The size of *r*_*ij*_ can be used to weigh the connection between node *i* and *j* [10]; the vector of node *i* is **i**, and the vector of node *j* is **j**.

## 3. Remotely Sensed Image Classification Approach Based on Complex Networks

For better cluster realization to remotely sensed image classification, overcoming sensitive shortcoming of the *K*-means [14, 15] algorithm to the initial cluster center, we first choose initial cluster center according to the eye measurement, then cluster center selection of each iteration follows the sorting of the nodes' WCF size, and the high node of WCF is taken as initial cluster center (the connection between cluster center later chosen and present cluster center should be smaller than the given threshold value *T*). Such cluster center selected has strong joint strength and strong local accumulation nature which is similar to other nodes, moreover, the probability of the same kind is relatively small among the cluster centers, which reduces the iteration times of the algorithm.

In this paper, a vector which is composed of the pixels from the same location of each band is considered as a node. The similarity between nodes is taken as the weighted degree, which represents the connected degree of these two nodes. In addition, the weak connected edge that the value of weighted degree is smaller than the threshold is deleted.

The Kappa coefficient here is used to measure the agreement between two raters who each classify *N* items into *C* mutually exclusive categories. The equation for Kappa coefficient (*κ*) is

(9) $$\kappa =\frac{\text{Pr}\left(a\right)-\text{Pr}\left(e\right)}{1-\text{Pr}\left(e\right)},$$

where Pr(*a*) is the relative observed agreement among raters, and Pr(*e*) is the hypothetical probability of chance agreement, using the observed data to calculate the probabilities of each observer randomly saying each category. If the raters are in complete agreement then  *κ* = 1. If there is no agreement among the raters other than what would be expected by chance (as defined by Pr(*e*)), *κ* = 0 [16].

Input: remotely sensed image.

Output: image classification, classified precision, Kappa coefficient.

**Step 1**

The hyperspectral image file is read and pretreated. These include radiation adjustment processing, geometry correction processing, mosaic processing, and cutting out processing [17, 18].

**Step 2**

The band data is chosen to do the experiment according to the standard deviation and corresponding coefficients.

**Step 3**

The weighted degree WD based on the formula (5) is calculated, clustering coefficient WC based on the formulas (5) and (6) is calculated, and complex network synthesis characteristic value WCF again based on the formula (7) is computed.

**Step 4**

The *k* maximal complex network synthesis characteristic values are chosen from the results computed in Step 3 by the top-k algorithm which chooses *k* maximal numbers using one-dimensional array.

**Step 5**

The connected degree between pixel nodes is calculated based on the formula (8).

**Step 6**

The threshold *T* of connected degree between pixel complex network nodes is computed by maximum mean square error.

**Step 7**

The connected degree between *k* selected nodes in Step 4 and initial cluster center is calculated, if the connected degree is smaller than threshold value *T*, then this node is taken as the cluster center.

**Step 8**

For the new cluster center, the distance between the sample and each new cluster center is computed and compared, and the sample is assigned to the class with the smallest distance.

**Step 9**

For the new class, the cluster center is recalculated. If the results are completely the same with the previous results, then the assorting process ends. Otherwise, turn to Step 3.

**Figure 1:**
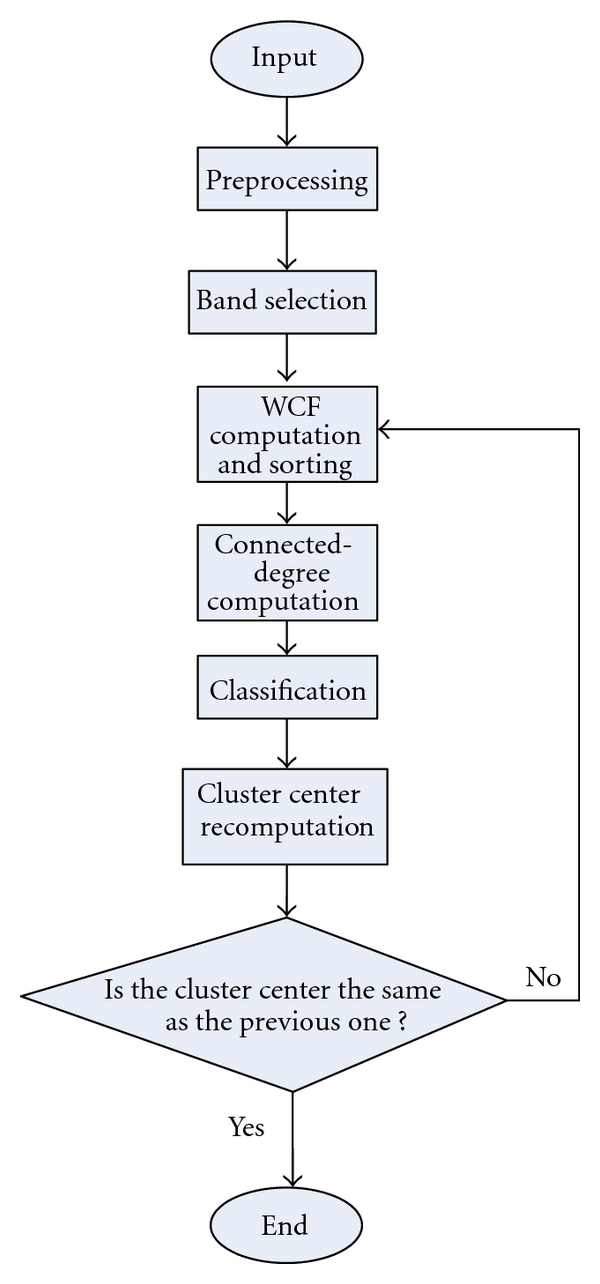
Flow chart of the proposed algorithm.

The proposed algorithm can be depicted by the following flow chart (Figure 1).

## 4. Experiments and Results Analysis

### 4.1. Simulated Images Experiments

These experiments are used to validate the accuracy of remotely sensed image classification method based on the eigenvalue and connected degree of complex network and compare the classification accuracy with *K*-means classification,ISODATA classification.

**Figure 2:**
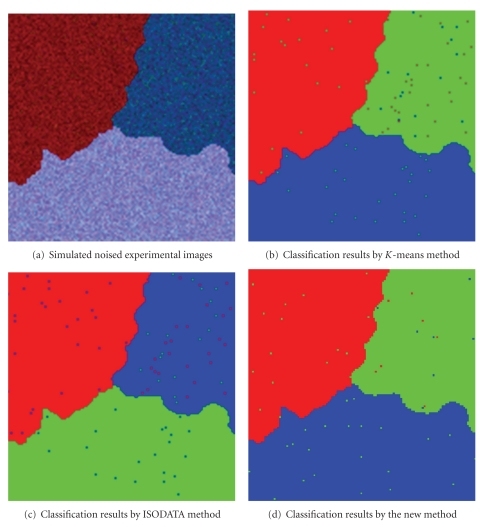
Classification results of simulated experimental images and different algorithms of three bands with added Gaussian noise.

The experiment designs simulated experimental images of three bands with Gaussian noise. Suppose the noise of each band is independent identically distributed Gaussian random noise, with zero mean and variance 0.01. The size of simulated experimental image is 128 × 128, and the noise image is shown in Figure 2(a). Figures 2(b), 2(c), and 2(d) are the results of the *K*-means classification method, the ISODATA classification method, and the new method, respectively. 

The classification accuracy percentage is firstly calculated, and the statistical results are shown in Table 1. 

From Table 1, for the entire sample data, it can be seen that the classification accuracy is up to 99.5% by *K*-means classification method, 99.4% by ISODATA classification method, and 99.6% by the new method based on complex network. The experiments show that the classification accuracy by the new method based on complex network is better than that of both traditional *K*-means classification method and ISODATA classification method.

The Kappa coefficient of classified result by the new method is computed below.

Confusion matrix is firstly calculated, and the results are shown in Table 2. Then the Kappa coefficient, Kappa error, and maximum possible Kappa are calculated by confusion matrix. The Kappa coefficient is 0.994 and 0.993 for the *K*-means classification method and ISODATA classification method, respectively (given the limited space of the paper, tables are omitted here).

From the relationship between Kappa coefficient and classified accuracy in Table 2, proposed method based on complex network is excellent.

### 4.2. AVIRIS Images Experiments

The classification accuracy of algorithms is very important. In order to obtain more accurate classification precision, the parts of AVIRIS hyperspectral data are selected in the experiments, which is photographed in a remote sensing experimental plot of the northeast of the US Indiana in June 12, 1992 [19], and the band number is 220, and data comes from the website http://engineering.purdue.edu/~biehl/MultiSpec.

In the high spectrum remotely sensed image selected by the experiments, we should select the bands that are less polluted by the moisture noise because some bands are polluted seriously by the moisture noise. Band selection [20, 21] mainly depends upon two essential factors, the standard deviation and the correlation coefficient. The larger the band standard deviation is, the more information this band contains. The larger the correlation coefficient between two bands is, the more information similarity the two bands contain. The band selection steps are as follows.

**Step 1**

First the standard deviation of each band is calculated and arranged in descent order, as shown in Table 3. Due to limited space, here we only give the partial data (first 30 bands).

**Step 2**

Calculate the correlation coefficients (CCs) between two bands, as shown in Table 4. Due to limited space, we only give the partial data.

For the band having quite great similarity, we only need to choose one of the bands, between two essential factors the standard deviation and the correlation coefficient of the bands influenced, and we should choose bands with the larger standard deviation and smaller correlation coefficient between each other.

**Step 3**

According to the standard deviation in Step 1 and the correlation coefficients in Step 2, we choose band 42, band 29, and band 120 to do the experiments based on the principle that the standard deviation is as large as possible and correlation coefficient is as small as possible.

In order to compare the classification precision, the new algorithm, which is a nonsupervised classification approach, is compared with other two nonsupervised classification approaches, the *K*-means algorithm and the ISODATA algorithm. Finally the precision of the three kinds of classification results is compared. In order to count the uniformity of the classified precision, the classification number of the three classification approaches is all set to 3. 

**Figure 3:**
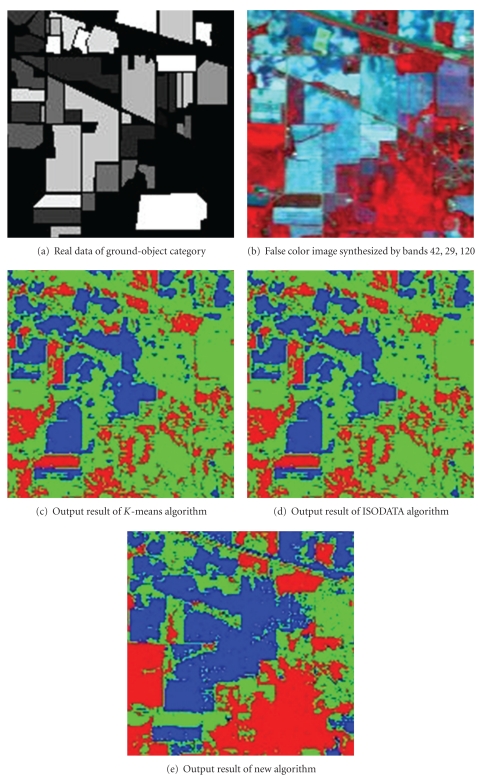
Raw remotely sensed data and classified results by different algorithms.

Figure 3(a) shows the real data of ground-object category, and Figure 3(b) shows the false color image synthesized by bands 42, 29, 120. The result with the *K*-means algorithm is shown in Figure 3(c). The result with the ISODATA algorithm is shown in Figure 3(d). The result with the new algorithm is shown in Figure 3(e), in which the value of *a* is set to 0.5.

 The classification accuracy of simulated experimental image by each classified method is counted below. In this experiment the image pixels are chosen as size of 145 × 145 to count overall classification precision of the remotely sensed image. We extract the subset with size of 42 × 145 to compute the classification precision. The statistical results are shown in Table 5. From Table 5, the classification result of the samples in category 1 by new algorithm is better than the *K*-means algorithm and the ISODATA algorithm; the result of the samples in category 2 by new algorithm is worse than the *K*-means algorithm and the ISODATA algorithm; the classification result of the samples in category 3 by new algorithm is much better than the *K*-means algorithm and the ISODATA algorithm. For all samples, the classified precision of the *K*-means algorithm achieves 82%, the classified precision of the ISODATA algorithm achieves 82%, but the classified precision of the new algorithm achieves 90.4%. The experiment shows that the classified precision of the new algorithm increases by 8%, compared to the traditional *K*-means algorithm and the ISODATA algorithm. 

The Kappa coefficient of the new algorithm based on complex network is calculated as follows: first the confusion matrix is calculated, which is shown as Table 6. In order to reduce the operand we only calculate the partial data. The three digits of the second line in Table 4 present the numbers of the samples which should originally belong to category 1 but are classified to category 1, category 2, and category 3, respectively. The three digits of the third line in Table 4 present the numbers of the samples which should originally belong to category 2 but are classified to category 1, category 2, and category 3, respectively. The three digits of the fourth line in Table 6 present the numbers of the samples which should originally belong to category 3 but are classified to category 1, category 2, and category 3, respectively. Using the confusion matrix already obtained we calculate the Kappa coefficient, Kappa error, and Maximum possible Kappa which is shown in Table 6. 

 The merits of this algorithm are illustrated as follows. This paper chooses well the initial cluster center according to the connection among nodes and the nodes' weighted network synthesis characteristic value and overcomes sensitive shortcoming of the *K*-means cluster algorithm to the initial value, thus, it enhances greatly the cluster quality. It is easy to find that the method in this paper is better than that of traditional methods either from the classified accuracy or the cluster stability.

- It overcomes the sensitive shortcoming of the *K*-means cluster algorithm to the initial value, which is easily fallen into the local region.
- According to the connection of the node, cluster center is selected, which reduces the probability of selecting different nodes of the same kind as the cluster center, reduced the iterative times of the algorithm, and raised the algorithm efficiency.

## 5. Conclusion

This paper proposes a remotely sensed image classification approach based on the complex network eigenvalue and connected-degree combining weighted complex network synthesis characteristic value with *K*-means cluster algorithm. Simulated experiments classify simulated experimental image of 3 bands with added Gaussian noise and AVIRIS hyperspectral remotely sensed data photographed in a remote sensing experimental plot of the northeast of the US Indiana in June 12, 1992, where the classified accuracy is improved compared to traditional *K*-means method and ISODATA method. Although the new method proposed carries on the classification well to the remotely sensed image, as a result of the own complexity of remotely sensed image, it needs deep research to choose the optimal characteristic and improve the accuracy of remotely sensed image classification.

## Figures and Tables

**Table 1:** Classification accuracy percentage of simulated images by three classification methods.

Project	Class 1	Class 2	Class 3	Classification
				accuracy
Ground truth	4402	5482	6500	100%
*K*-means classification method	4359	5461	6474	99.5%
ISODATA classification method	4359	5455	6478	99.4%
New method based on complex network	4387	5467	6477	99.6%

**Table 2:** Confusion matrix of classified results by proposed method based on complex network.

Project	Class 1	Class 2	Class 3
Class 1	4387	7	8
Class 2	15	5467	0
Class 3	23	0	6477
Kappa coefficient	0.9951		
Kappa error	0.0007		
Maximum Possible Kappa	0.9979		

**Table 3:** First 30 bands and standard deviation descending according to the standard deviation.

Band	Standard deviation	Band	Standard deviation	Band	Standard deviation
Band 29	1012.186414	Band 33	866.810154	Band 50	771.726478
Band 28	995.72999	Band 23	864.978506	Band 20	764.574815
Band 27	932.214293	Band 31	856.363198	Band 45	757.351603
Band 26	932.1544	Band 43	853.999667	Band 51	755.848178
Band 25	910.697106	Band 22	839.800843	Band 52	754.248011
Band 30	908.840288	Band 44	837.885813	Band 34	742.718947
Band 42	907.544127	Band 39	807.049573	Band 19	731.861102
Band 32	898.713883	Band 21	797.873879	Band 53	727.076556
Band 41	884.337735	Band 48	788.812468	Band 38	720.686685
Band 24	875.553739	Band 49	775.506589	Band 47	719.547723

**Table 4:** The correlation matrix of partial band.

	CCs	Band								
		Band 29	Band 30	Band 31	Band 32	Band 33	Band 34	Band 35	Band 36	Band 37
Band	Band 29	1	0.99	0.99	0.99	0.99	0.96	0.87	0.47	−0.16
	Band 30	0.99	1	0.99	0.99	0.99	0.97	0.88	0.47	−0.15
	Band 31	0.99	0.99	1	0.99	0.99	0.98	0.91	0.51	−0.12
	Band 32	0.99	0.99	0.99	1	0.99	0.97	0.88	0.47	−0.15
	Band 33	0.99	0.99	0.99	0.99	1	0.98	0.91	0.52	−0.11
	Band 34	0.96	0.97	0.98	0.97	0.98	1	0.96	0.63	0.01
	Band 35	0.87	0.88	0.91	0.88	0.91	0.96	1	0.81	0.26
	Band 36	0.47	0.47	0.51	0.47	0.52	0.63	0.81	1	0.77
	Band 37	−0.16	−0.15	−0.12	−0.15	−0.11	0.01	0.26	0.77	1

*Note*. In Table 3 the standard deviation of band 29 is the greatest. In order to express the characteristics of correlation coefficients, the band listed in Table 4 begins with band 29.

**Table 5:** Classification statistics of partial data.

Item	Category 1	Category 2	Category 3	Classified
				precision
Ground data	966	3446	1678	100%
*K*-means	423	3525	2142	82%
ISODATA	418	3487	2185	82%
New algorithm based on complex network	1251	3153	1684	90.4%

**Table 6:** Confusion matrix of classification results by new algorithm based on complex network.

Item	Category 1	Category 2	Category 3
Category 1	883	315	139
Category 2	192	2377	121
Category 3	176	461	1424
Kappa Coefficient	0.6353		
Kappa error	0.0085		
Maximum possible Kappa	0.8797		
